# The “Data Visualization Clinic”: a library-led critique workshop for data visualization

**DOI:** 10.5195/jmla.2018.333

**Published:** 2018-10-01

**Authors:** Fred Willie Zametkin LaPolla, Denis Rubin

**Affiliations:** Knowledge Management Librarian, NYU Health Sciences Library, New York University, New York, NY; Lead Quantitative/Statistical Data Analysis Specialist, NYU Data Services, New York University, New York, NY

## Abstract

**Background:**

The authors’ main university library and affiliated academic medical center library sought to increase library programming around data visualization, a new service area for both libraries. Additionally, our institution is home to many researchers with a strong interest in data visualization but who are generally working in isolation of one another.

**Case Presentation:**

This case study describes an innovative workshop, the “Data Visualization Clinic,” where members of our library’s community bring in data visualization projects such as figures in papers, projects hosted online, and handouts and receive constructive feedback from a group of peers. The authors detail the process of hosting a clinic and the feedback that we received from participants.

**Conclusions:**

The “Data Visualization Clinic” offers a viable workshop to leverage expertise of library users and build the library’s reputation as a hub of data visualization services without heavy investment in infrastructure like special monitors or coding skills. That said, it faces the challenge of relying on the participation of the broader community, which is often pressed for time. The event can also serve as an opportunity for researchers who have an interest in data visualization to meet and network.

## BACKGROUND

As academic and health sciences libraries have increasingly moved into providing services in data visualization [1–15], it is important to find innovative ways to provide relevant visualization-related programming for library patrons. In the realm of health sciences libraries, scholarly literature and library websites describe workshops and classes that have often been of a didactic educational nature or a hands-on instructional workshop. These have varied from both for-credit and not-for-credit introductions to data visualization best practices [8, 13, 16] to skills-based workshops teaching technical tools like R and Cytoscape [2–5, 10–15].

Additionally, several institutions hold events with presenters speaking on a project or visualization topic of interest to them. For example, Duke University Library hosts the Duke Visualization Forum Friday, a series of talks held by members of the Duke community on their data visualization development and application research. Events at Duke include showcasing a project aimed at visualizing the neuron and a visualization project on human populations for public health [9]. Similarly, North Carolina State University Libraries host a “Coffee and Viz” series, where experts at their institution speak on a given visualization-related topic. Examples of past events at North Carolina State University’s Coffee and Viz have included topics of visualizing the solar system and visualizing electron models [10]. These workshops allow experts in data visualization to discuss ongoing and recently completed projects with the broader community.

While educational, instructor-led classes provide patrons with new skills, they do require significant dedication of a librarian’s time in terms of researching and organizing content in order to provide a successful workshop and provide limited time for students to apply what they have learned to their own projects. An alternative pedagogical option that allows students to receive feedback on their own work is a critique method, wherein students present designs and receive feedback from their peers [17]. A critique model has the advantage of allowing the designers to learn by getting feedback and move beyond their own perspectives on their work, and it allows other participants to learn by observing and evaluating what does and does not work and by moving through structured stages of interrogating a design, reflecting on it, and articulating critical feedback [17].

Academic institutions and academic medical centers both have large numbers of researchers and students with an interest in data visualization who can serve as an ideal pool for critique sessions. Critique sessions differ from offerings like those at Duke and North Carolina in that the workshops span multiple projects, often focusing primarily on figures for a paper or poster rather than a visualization project per se and do not have a preannounced speaker talking on one specific topic [9, 10]. Instead members, of the university and academic medical center community are invited to join, and multiple projects can be quickly discussed in a single session.

Beginning in the spring of 2016, our university academic library began hosting an event, called the “Data Visualization Clinic,” for users to present and receive feedback and critiques on data visualizations ranging from digital humanities projects to figures for scientific papers and posters. This event is novel in that it provides an opportunity for our university community members to showcase visualizations that are still in development and to receive constructive feedback, typically with the goal of improving publications but also frequently for quality improvement (QI) reporting and dashboard creation. These events do not entail data visualization experts presenting on a finished project but rather allow researchers in other specialties to receive feedback on visualizations that they are developing. Seeing the interest generated by the events at our main university library, our health sciences library (located on a separate campus from the main library) decided to host its own “Data Visualization Clinic,” oriented primarily for medical center patrons.

## CASE STUDY PURPOSE

The purpose of this case study is to provide an overview of the “Data Visualization Clinic,” a critique workshop [17] for community members in an academic library setting. The clinics offer a low-cost, low-barrier-to-entry method to leverage the expertise and enthusiasm of the community of library patrons, while at the same time making use of the library’s role as a hub for researchers. This case study will describe the format, outreach, logistics, and outcomes of clinics held at the academic library’s main library and connected health sciences library.

## CASE PRESENTATION

### Format

The “Data Visualization Clinic” follows a discussion-oriented workshop format, along the lines of a critique workshop [17]. Before the session begins, users who register online are asked in the sign-up form if they will submit a visualization for feedback, and those who agree are contacted by the library to submit a project. Examples of visualizations include charts for a paper or poster, handouts, or dashboards of physician performance in a given department.

The majority of attendees tend not to submit a project but attend solely as audience members to provide feedback. Our participants are typically primarily working in a research specialization, such as emergency medicine or European history, and are looking to improve their charts and projects rather than researchers focused on data visualization in and of itself. The projects typically take the form of images that can be added to a PowerPoint presentation or uniform resource locators (URLs) for interactive online visuals.

The events’ organizers begin each session by providing an explanation of the rules and expectations for the event, followed by a brief overview of some general data visualization best practices that lasts for about ten minutes (typically selected to reflect topics relevant to that day’s session). Participants who submitted visuals are then called up to present on what they have done for ten to fifteen minutes (the time can vary based on how many people are attending the hour-and-a-half session).

Participants are invited to show their visualizations, explain their goals, explain the general context of the project (e.g., its topic, whether the visualization is related to a research paper), discuss the intended format and audience, and point out areas for which they would like feedback. We invite all members of our community to participate without regard to faculty, staff, or student role. The ten to fifteen minutes includes time for the “Data Visualization Clinic” audience to ask questions and provide feedback on what they feel works well and what could be improved about a project. The organizers keep time, facilitate moving from one presenter on to the next, and offer their own views on the work.

### Outreach

Several methods of outreach are employed to market the clinics. Events are promoted on both libraries’ websites. Emails are sent to library liaison groups as well as individuals who have taken past library classes and workshops in data visualization–related topics. When feasible, liaison librarians mention the clinics in person to their liaison groups during presentations, such as at a search session for a given department. At the medical center, three clinics overlapped with a series of data science–related classes [16], and promotion for the events was combined, allowing the “Data Visualization Clinic” to “piggy-back” on promotions for those classes. Also clinics are promoted at the medical center on an internal intranet portal’s news section.

### Logistics

“Data Visualization Clinic” workshops have ranged from two to six presenters, and users have typically been allotted ten to fifteen minutes, though with no requirement to fill that time if they finish beforehand. The events are held in a library classroom equipped with a large monitor or projector. The vast majority of projects are displayed in PowerPoint format, with some displayed in web browsers, and the clinics use technology that is already available in the library.

The major costs for the events entail time contacting participants to get visuals together for the events, preparation of relevant examples to promote discussion, email marketing and outreach, and the cost of snacks, which have typically been coffee and pastries and which are not, strictly speaking, required for the events.

In past clinics, the majority of people attended as spectators. That said, they are active participants in that they provide their insights and expertise on work being presented, which requires development of critical thinking skills and articulation of views regarding both aesthetics and efficacy of projects [17].

### Outcomes

To date, there have been 9 “Data Visualization Clinics” held at our main university library with 181 registered individuals. The largest volume of people came from the Physics Department, representing 6.63% of the total population, but most attendees were the sole representative of their departments, with 115 registered as a unique member of a department. At 4 clinics in our medical library, 57 individuals have attended, 10 of whom were repeat attendees. Sixteen visualizations were presented at the medical library. The largest volume of people came from departments where we have active liaison outreach, such as Population Health and Radiology, but other departments like Patient Experience Evaluation were heavily represented mostly due to interest among members of that community, as they did not have direct liaison support from the library. Supplemental Appendix A: “Roles and Departmental Affiliations of Attendees” provides a detailed breakdown of attendance by role and department.

Clinic attendance has ranged from 5–16 individuals at the medical center and from 5–25 at the main library. We were surprised to have representation from departments that are not conducting clinical or scientific research, in particular our hospital engineering department, which was trying to get feedback on how non-engineers would view a handout on a new construction project. Regarding participant roles, faculty and students attended, but uptake was particularly strong among research staff such as research coordinators, of whom there were 9 or nearly 16% of all participants. This surprised us and revealed a blind-spot for the library, as previously most of our outreach had been aimed at faculty and students.

Projects tended to be made using Microsoft Office tools, such as Excel and PowerPoint, although a handful used R, Tableau dashboards, or some other tool. Presenters did not have to disclose what software programs they used, so no hard figures are available on methods of image generation. In general, the presentations were primarily about design and comprehension rather than technology. This allowed more people to participate, as no background in a given tool or coding language was required. Librarians in our organization have a light familiarity with R, Tableau, and other tools, which at times has proved helpful, but the primary focus of the “Data Visualization Clinics” has never been on providing advice of a technical nature. Additionally, because images were presented on a large classroom computer monitor using PowerPoint or a web browser, special equipment was not required. While our classroom has a large computer monitor, a projector connected to a computer would serve the same purpose.

At the main university library, 64 attendees completed and returned an evaluation form (supplemental Appendix B: “Academic Library Evaluation Template”), with 95% indicating that they learned something new and useful about visualization and 82% expressing an interest in bringing a visualization for discussion next time. The majority of the free-form comments were positive and complementary of the open discussion format and interactivity of the session, although a number of people expressed a desire for a hands-on component for learning visualization tools.

At the medical center, of the 55 individuals who completed an evaluation form, 87% said they would either highly recommend or recommend the session (supplemental Appendix C: “Health Sciences Library Evaluation Template”). Most individuals said they had either learned a great deal (42%) or a moderate amount (42%), representing the highest and second highest rankings. Half (50%) of the individuals said that they would be willing to submit a visualization for discussion next time.

In the free-text responses to a question asking what the attendees hoped to get out of the sessions, responses could be roughly divided into comments seeking the feedback of other participants (35%) and looking for insights on new techniques of visualization or how to better visualize their data (52%), with the remainder leaving the question blank. Two respondents indicated that they had not anticipated a discussion-driven session and had wanted more educational content.

With regard to overall impressions at the medical center, 48% wrote generally positive feedback expressing enjoyment of the experience, and 25% noted appreciation for the community-building aspect of the event.

## DISCUSSION

The “Data Visualization Clinic” has offered a way to build community and provide library programming around data visualization. Participants in general have been enthusiastic about the experience and have been willing to foster discussion in our sessions. The clinic represented a low-cost opportunity, because, aside from refreshments and time spent organizing the event, little needed to be invested up front. Moreover, because the event was participant-driven, it was not necessary to develop a session of educational material. We believe that this event is an adaptable opportunity to develop a library’s role as a data visualization hub.

That said, the major challenge has been to consistently get a large enough group to attend either as audience or participants. We have held the event with as few as five individuals attending, but the discussions have tended to be better with a larger group. There have also been two instances of events being cancelled due to lack of enrollment. Unfortunately, to date, no good solution for this problem exists. Anecdotal observation indicates that certain times of the year and the week work better than others, though the number of workshops held is too small and the idiosyncrasies of planning have too many confounding factors to draw any meaningful conclusions or inferences. With that caveat in mind, it would appear to us that holding events on Friday or Thursday mornings at less busy times of the year encourages larger groups, although if it is at a time when many people are on vacation (such as August), it can be hard to fill the room. This problem is further compounded in that many individuals are busy and will choose a more pressing work commitment over a non-required library event.

Tying promotion to a larger event also seems to help, but in general, marketing of events is one of the major challenges that our library faces. While experimentation in methods of promoting classes and workshops is ongoing, to date no obvious solution has presented itself aside from trying to promote events widely and in multiple formats. These problems are not unique to the “Data Visualization Clinic,” and marketing and attendance are a challenge in most workshops we offer. That said, due to the critique-design of the clinics, low turnout is a particular obstacle to success.

Another challenge has been that occasionally presenters have not carefully read through the event description or have interpreted the description in a way that was contrary to our intended meaning and, thus, were surprised that they had to present to a room of peers. This outcome has only happened once in our experience, and while the presenter was surprised, she ultimately was willing to present to and receive feedback from the group.

An additional challenge that has presented itself a handful of times has been individuals seeking help of primarily a technical nature. These have included a desire for information on how to use R and other coding languages to create a desired effect. Because neither the librarians leading the event nor the attendees may have the requisite subject matter or technical expertise, typically this leads to a lack of conversation. In these cases, we have found it to be helpful to state clearly that neither we nor the rest of the room seems to be able to offer help and we move on to the next presentation.

These challenges can be frustrating, and no obvious solution has presented itself to date. That said, in general, the events have been enjoyable and the comments, both in our paper evaluations and by individuals, seem to indicate that the “Data Visualization Clinics” are helpful to our patrons. Moreover, by fostering conversations between library users, we believe we are leveraging the library’s role as a hub for research to improve research communications and possibly to allow new collaborations and ideas to form.

## SUPPLEMENTAL FILES

Appendix A Roles and departmental affiliation of attendees

Appendix B Academic library evaluation template

Appendix C Health Sciences Library evaluation template
