# Supplementary material for: The “Data Visualization Clinic”: a library-led critique workshop for data visualization
Source: J Med Libr Assoc. 2018 Oct 1;106(4):477–82. doi: 10.5195/jmla.2018.333 (PMC6148617; doi:10.5195/jmla.2018.333)
Supplement: Appendix A [file jmla-106-477-s001.pdf]

JMLA Journal of the Medical Library Association

SC

Supplemental content to
J Med Libr Assoc. Oct;106(4):dx.doi.org/10.5195/jmla.2018.333
www.jmla.mlanet.org
© LaPolla, Rubin 2018

The "Data Visualization Clinic": a library-led critique workshop for data visualization

Fred Willie Zametkin LaPolla; Denis Rubin

APPENDIX A

Roles and departmental affiliation of attendees

Medical Center

Division/Department	Count	Percentage	Role	Count	Percentage
Care Management	2	3.51%	Administrator	3	5.26%
Child Study Center	1	1.75%	Analyst	11	19.30%
Clinical Informatics	1	1.75%	Assistant Director of Finance and Analytics	1	1.75%
Clinical Nurse Informatics	1	1.75%	Clinical Nurse Informaticist	2	3.51%
Developmental Genetics	1	1.75%	Digital Designer User Interface and User Experience	1	1.75%
Digital Solutions	2	3.51%	Faculty	7	12.28%
Emergency Medicine	3	5.26%	Health Information Specialist	1	1.75%
Fala Institute	1	1.75%	Informant Volunteer	1	1.75%
Medical Education	1	1.75%	Intern	2	3.51%
Library	1	1.75%	Manager	2	3.51%
Neuroscience	2	3.51%	Nursing Research Program Coordinator	1	1.75%
Nursing	2	3.51%	Patient Experience Designer	2	3.51%
Nursing Informatics	1	1.75%	Patient Experience Improvement Specialist	1	1.75%
Office of Science and Research	1	1.75%	PhD Student	1	1.75%
Pathology	2	3.51%	Post Doc	2	3.51%
Patient Experience	10	17.54%	Program Manager	2	3.51%
Patient Family Education	1	1.75%	Research Coordinator	9	15.79%
Population Health	16	28.07%	Research Data Associate	2	3.51%
Psychiatry	1	1.75%	Scientist	1	1.75%
Radiology	3	5.26%	Staff	1	1.75%
Engineering/Construction	1	1.75%	Student	4	7.02%
School of Medicine	1	1.75%	Total		100.0%
Blank	2	3.51%			
Total		100.0%			

Supplemental content to
J Med Libr Assoc. Oct;106(4):dx.doi.org/10.5195/jmla.2018.333
www.jmla.mlanet.org
© LaPolla, Rubin 2018

Main Campus

Department/Division	Count	Percentage	Role	Count	Percentage
Physics	12	6.63%	Masters	80	44.20%
Integrated Marketing	7	3.87%	Staff or Administrator	36	19.89%
MPA	5	2.76%	Doctoral	21	11.60%
Management of Technology	4	2.21%	Faculty	19	10.50%
Industrial Engineering	3	1.66%	Other	9	4.97%
MASY	3	1.66%	Alumni	9	4.97%
Psychology	3	1.66%	Postdoc	2	1.10%
Cinema Studies	3	1.66%	Senior	2	1.10%
.	2	1.10%	Junior	2	1.10%
Applied Statistics	2	1.10%	MaRLI	1	0.55%
Electrical Engineering	2	1.10%	Total	181	100.00%
English	2	1.10%			
Food Studies	2	1.10%			
Health Sciences Library	2	1.10%			
MOT	2	1.10%			
MPA-PNP	2	1.10%			
MSCS	2	1.10%			
MSIS	2	1.10%			
N/A	2	1.10%			
Open Arts	2	1.10%			
Computer Science	2	1.10%			
Academic Affairs	1	0.55%			
Academic Programs	1	0.55%			
Advanced Diploma in Monitoring and Evaluation	1	0.55%			
Anthropology	1	0.55%			
Applied Quantitative Research	1	0.55%			
AQR	1	0.55%			
Art History/Library Science	1	0.55%			
Bioinformatics	1	0.55%			
Budget and Planning	1	0.55%			
Cardiology and Comprehensive Care	1	0.55%			
Center for Academic Excellence and Support, Office of Educational Technology	1	0.55%			

SC
Supplemental content to
J Med Libr Assoc. Oct;106(4):dx.doi.org/10.5195/jmla.2018.333
www.jmla.mlanet.org
© LaPolla, Rubin 2018

Department/Division	Count	Percentage	Role	Count	Percentage
Center for Leadership and Human Capital Management	1	0.55%			
Center for Neural Science	1	0.55%			
Center for Urban Science and Progress	1	0.55%			
Chemistry PhD	1	0.55%			
Clinical Social Work	1	0.55%			
CNS PhD	1	0.55%			
Cognition and Perception	1	0.55%			
Community Affairs	1	0.55%			
Comparative Literature	1	0.55%			
Computational Biology/Mathematics	1	0.55%			
Computer Engineering	1	0.55%			
Continuing Ed	1	0.55%			
Data Visualization	1	0.55%			
Data Visualization Clinic	1	0.55%			
Data Visualization Clinic No. 7	1	0.55%			
Data Visualization Clinic No.8	1	0.55%			
Decision Support Group	1	0.55%			
Department of Population Health	1	0.55%			
Department of Population Health, Section for Health Equity	1	0.55%			
Digital Studio	1	0.55%			
Digital Studio, TLT	1	0.55%			
Digital Studio/TLT	1	0.55%			
East Asian	1	0.55%			
Economics	1	0.55%			
Education Technology	1	0.55%			
Educational Technologist	1	0.55%			
Elearning Services	1	0.55%			
EMPA	1	0.55%			
EMPA Public Leaders	1	0.55%			
Engineering	1	0.55%			

Department/Division	Count	Percentage	Role	Count	Percentage
FAS	1	0.55%			
Gcmt	1	0.55%			
Global Affairs	1	0.55%			
Global Exchange	1	0.55%			
Grad	1	0.55%			
Graduate	1	0.55%			
Graduate From Tisch	1	0.55%			
Health Policy & Management	1	0.55%			
HRMD	1	0.55%			
IDM	1	0.55%			
IE	1	0.55%			
IHDSC	1	0.55%			
IM	1	0.55%			
International Education	1	0.55%			
International Political Business	1	0.55%			
International Relations	1	0.55%			
IO Psychology	1	0.55%			
IR	1	0.55%			
Journalism	1	0.55%			
Liberal Studies	1	0.55%			
Libraries	1	0.55%			
Library	1	0.55%			
M.S in Integrated Marketing	1	0.55%			
M.S. Urban Science and Informatics	1	0.55%			
MA in Food Studies	1	0.55%			
MA in Political Science	1	0.55%			
Management of Technology and Innovation	1	0.55%			
Management of Technology for Executives	1	0.55%			
Manufacturing Engineering	1	0.55%			
Master Science of Integrated Marketing	1	0.55%			
Masters in Computer Science	1	0.55%			
Masters of Applied Urban Science and Informatics	1	0.55%			
MBA	1	0.55%			

Supplemental content to
J Med Libr Assoc. Oct;106(4):dx.doi.org/10.5195/jmla.2018.333
www.jmla.mlanet.org
© LaPolla, Rubin 2018

Department/Division	Count	Percentage	Role	Count	Percentage
Mcghee	1	0.55%			
Mcghee Division	1	0.55%			
Media, Culture, and Communication	1	0.55%			
Medical Librarian	1	0.55%			
Mobile AR Lab	1	0.55%			
MS in CE	1	0.55%			
MS in Data Science	1	0.55%			
MS in Information Systems	1	0.55%			
MS in Publishing	1	0.55%			
NA	1	0.55%			
Nutrition and Dietetics	1	0.55%			
NYU College of Global Public Health	1	0.55%			
NYU IT	1	0.55%			
NYU IT TLT	1	0.55%			
NYU Silver	1	0.55%			
NYU Steinhardt	1	0.55%			
NYU Stern	1	0.55%			
Office of Educational Technology	1	0.55%			
Ph.D.	1	0.55%			
PhD	1	0.55%			
Philanthropy	1	0.55%			
Political Science	1	0.55%			
Politics	1	0.55%			
Post-Doc	1	0.55%			
PRCC	1	0.55%			
Public & Nonprofit Management & Policy	1	0.55%			
Public Relations and Corporate Communication	1	0.55%			
Public Relations and Corporate Communications	1	0.55%			
Research	1	0.55%			
Social Work	1	0.55%			
Sociocultural Anthropology	1	0.55%			
SPS - IM	1	0.55%			

JMLA Journal of the Medical Library Association

Supplemental content to
J Med Libr Assoc. Oct;106(4):dx.doi.org/10.5195/jmla.2018.333
www.jmla.mlanet.org
© LaPolla, Rubin 2018

Department/Division	Count	Percentage	Role	Count	Percentage
Steinhardt Technology Services	1	0.55%			
Stern	1	0.55%			
Student Affairs	1	0.55%			
Teaching and Learning Technology	1	0.55%			
Urban Informatics	1	0.55%			
Urban Science and Informatics	1	0.55%			
Urban System Engineering and Management	1	0.55%			
Wasserman	1	0.55%			
Wasserman Center for Career Development	1	0.55%			
Total		100.0%			
