# Supplementary material for: The “Data Visualization Clinic”: a library-led critique workshop for data visualization
Source: J Med Libr Assoc. 2018 Oct 1;106(4):477–82. doi: 10.5195/jmla.2018.333 (PMC6148617; doi:10.5195/jmla.2018.333)
Supplement: Appendix B [file jmla-106-477-s002.pdf]

JMLA Journal of the Medical Library Association

SC

Supplemental content to
J Med Libr Assoc. Oct;106(4):dx.doi.org/10.5195/jmla.2018.333
www.jmla.mlanet.org
© LaPolla, Rubin 2018

The "Data Visualization Clinic": a library-led critique workshop for data visualization

Fred Willie Zametkin LaPolla; Denis Rubin

APPENDIX B

Academic library evaluation template

What motivated you to come to this session?

What data visualization tools have you used in the past?

Have you learned anything new and useful about visualization? ☐ Yes ☐ No
Please tell us:

Would you bring a visualization to discuss next time? ☐ Yes ☐ No

Is there anything about the format that you liked?

What about the format would you improve?

What else would you like to see during this session?

What did you think of our reference page guides.nyu.edu/viz? Do you have any suggestions for improvement?

Do you have any other suggestions for improving this session?
