# Supplementary material for: The “Data Visualization Clinic”: a library-led critique workshop for data visualization
Source: J Med Libr Assoc. 2018 Oct 1;106(4):477–82. doi: 10.5195/jmla.2018.333 (PMC6148617; doi:10.5195/jmla.2018.333)
Supplement: Appendix C [file jmla-106-477-s003.pdf]

JMLA Journal of the Medical Library Association

Supplemental content to
J Med Libr Assoc. Oct;106(4):dx.doi.org/10.5195/jmla.2018.333
www.jmla.mlanet.org
© LaPolla, Rubin 2018

The "Data Visualization Clinic": a library-led critique workshop for data visualization

Fred Willie Zametkin LaPolla; Denis Rubin

APPENDIX C

Health Sciences Library evaluation template

School:
- School of Medicine
- College of Dentistry
- College of Nursing
- Tisch Hospital
- Other:

Department/Division:

What is your role (e.g., postdoc, faculty, student, intern, administrator)?

What did you hope to get out of this event and to what degree did you get that?

Would you recommend this clinic to others?
- Highly recommend
- Recommend
- Recommend with reservations
- Not recommend

Do you feel that you gained a better understanding of data visualization?
- Yes a great deal better understanding
- Yes a moderate amount of better understanding
- Yes a little bit of understanding
- No, no improvement

Do you feel that this event will be helpful to creating your own visualizations:
- It was very helpful
- It was slightly helpful
- It was slightly unhelpful
- It was very unhelpful

JMLA Journal of the Medical Library Association

SC

Supplemental content to
J Med Libr Assoc. Oct;106(4):dx.doi.org/10.5195/jmla.2018.333
www.jmla.mlanet.org
© LaPolla, Rubin 2018

Do you feel interested in working with other participants in a data viz community of practice?

- o Yes
- o No

Would you be willing to submit your own visualizations for discussion in a Data Viz Clinic?

- o Yes
- o No

If yes, what is your email?

What are your overall impressions of the Data Visualization Clinic and how might it be improved?

Participants only: Was this experience useful to you, and how can it be improved?
